# TFSNet: A Time–Frequency Synergy Network Based on EEG Signals for Autism Spectrum Disorder Classification

**DOI:** 10.3390/brainsci15070684

**Published:** 2025-06-25

**Authors:** Lijuan Shi, Lintao Ma, Jian Zhao, Zhejun Kuang, Sifan Wang, Han Yang, Haiyan Wang, Qiulei Han, Lei Sun

**Affiliations:** 1 College of Electronic Information Engineering, Changchun University, Changchun 130022, China; shilj@ccu.edu.cn (L.S.); 230401176@mails.ccu.edu.cn (L.M.);; 2 Jilin Provincial Key Laboratory of Human Health Status Identification Function & Enhancement, Changchun 130022, China; 3 Key Laboratory of Intelligent Rehabilitation and Barrier-Free for the Disabled, Changchun University, Ministry of Education, Changchun 130012, China; 4 College of Computer Science and Technology, Changchun University, Changchun 130022, China

**Keywords:** Autism Spectrum Disorder, EEG, time-frequency feature fusion, feature extraction

## Abstract

Autism Spectrum Disorder (ASD) seriously affects social, communication, and behavioral functions, and early accurate diagnosis is crucial to improve the prognosis of patients. Traditional diagnosis methods rely on professional doctors to make subjective diagnosis through scales, the feature extraction of existing machine learning methods is inefficient, and existing deep learning methods have limitations in capturing time-varying features and the joint expression of time–frequency features. To this end, this study proposes a time–frequency synergy network (TFSNet) to improve the accuracy of ASD EEG signal classification. The proposed Dynamic Residual Block (TDRB) was used to enhance time-domain feature extraction; Short-Time Fourier Transform (STFT), convolutional attention mechanism, and transformation technology were combined to capture frequency-domain information; and an adaptive cross-domain attention mechanism (ACDA) was designed to realize efficient fusion of time–frequency features. The experimental results show that the average accuracy of TFSNet on the University of Sheffield (containing 28 ASD patients and 28 healthy controls) and KAU dataset (containing 12 ASD patients and five healthy controls) reaches 98.68%and 97.14%, respectively, yielding significantly better results than the existing machine learning and deep learning methods. In addition, the analysis of model decisions through interpretability analysis techniques enhances its transparency and reliability.

## 1. Introduction

Autism Spectrum Disorder (ASD) is a complex neurodevelopmental condition characterized by challenges in social interaction, communication, and repetitive behaviors [1,2]. The World Health Organization estimates that approximately 1 in 100 children worldwide are diagnosed with ASD, with prevalence varying significantly by region and socioeconomic status, reflecting global differences in diagnostic capabilities and service provision [3]. The etiology of ASD remains unclear, though research suggests it results from complex interactions between genetic and environmental factors [4,5].

Currently, ASD diagnosis relies on clinical observations and behavioral assessments by medical professionals [6,7,8,9], a process that is time-consuming and lacks objective biological indicators, potentially leading to diagnostic variability [10]. In recent years, an increasing number of studies have focused on identifying ASD through biomarkers, especially the electroencephalogram (EEG) [11]. While magnetic resonance imaging (MRI) [12] and computed tomography (CT) [13] are also used in ASD research, EEG offers advantages, including cost-effectiveness, high temporal resolution, and noninvasiveness [14]. Although EEG signal analysis requires preprocessing for noise and artifact removal [15], constructing ASD diagnostic models using EEG signals remains crucial for clinical screening and diagnosis support.

Many researchers are applying machine learning approaches to ASD EEG signal analysis for feature extraction and classification [16]. However, traditional machine learning methods typically rely on manual feature extraction, requiring tedious feature engineering that depends heavily on researcher experience and domain knowledge [17,18]. To address these limitations, researchers have explored increasingly complex feature extraction methods to improve classification performance [19,20,21].

The development of deep learning has made it a prominent area of research in ASD EEG signal analysis [22,23,24,25]. Compared to traditional machine learning, deep learning automatically extracts complex nonlinear features from data, greatly reducing the need for manual feature engineering. Researchers are continuing to explore methods for automating feature extraction and minimizing manual intervention. Tawhid et al. [26] proposed an effective diagnostic framework based on EEG spectrogram images. The authors applied a Short-Time Fourier Transform to convert the processed signals into 2D spectrogram images, which were then analyzed using machine learning. Ari et al. [27] proposed a method combining the Douglas–Peucker algorithm, sparse coding-based feature mapping, and a convolutional neural network. The authors sparsely encode the EEG rhythm during feature extraction and input it into a CNN for training. Baygin et al. [28] proposed a method for automatically detecting ASD. They extracted one-dimensional Local Binary Pattern (1D-LBP) features from electroencephalogram (EEG) signals and generated spectrogram images using Short-Time Fourier Transform (STFT). Pretrained MobileNetV2, ShuffleNet, and SqueezeNet models were used to extract deep features. Feature selection was performed using a two-layer ReliefF algorithm, resulting in excellent performance. Ardakani et al. [29] divided the EEG signals from autistic and healthy individuals into nonoverlapping windows, treated them as images, and classified them using a 2D deep convolutional neural network (2D-DCNN). Wadhera et al. [30] employed transfer learning to classify autism using pretrained EEGNet and DeepConvNet models, achieving accuracies of 89.6% and 92.3%, respectively. Ullah et al. [31] proposed a deep learning-based weighted ensemble model for classifying Autism Spectrum Disorder (ASD) using electroencephalogram (EEG) data. They converted the original EEG signals into multi-channel two-dimensional spectrograms using Short-Time Fourier Transform (STFT). A grid search strategy was then employed to determine the optimal model weight combination, resulting in excellent performance.

Despite recent advances in feature extraction research for autism signal classification, traditional convolutional neural networks are still insufficient to capture temporal variations in EEG signals. Due to the use of a fixed convolutional kernel, it is difficult for these models to adapt to the dynamic changes of EEG signals. In addition, most current studies lack a strategy to jointly represent time- and frequency-domain EEG signals at the feature level, which limits the effective capture of integrated signal information. These limitations lead to a weak generalization ability of existing methods when faced with different datasets in subject-related experiments, making it difficult to maintain stable performance under different acquisition environments and participant characteristics. To address these issues, we propose a time–frequency synergy network (TFSNet) that fuses time- and frequency-domain features for the classification of EEG signals in autism. This network introduces dynamic convolution into the time-domain feature extraction, which can adaptively adjust the parameters of the convolution kernel and enhance the ability to capture the temporal variations of EEG signals. In addition, this paper proposes an attention mechanism that fuses time-domain and frequency-domain features, which reveals the dynamic changes and complex patterns of EEG signals in greater depth while effectively representing the time-domain and frequency-domain features jointly. The contributions of this paper are as follows:

- A time–frequency synergy network model is proposed in this paper for the feature extraction and classification of autism EEG signals. This model effectively extracts key information from EEG signals and significantly improves classification performance.
- This paper introduces dynamic convolution into EEG time-domain feature extraction for ASD. We propose the TDRB module, which adaptively adjusts convolution kernel parameters and enhances the model’s ability to capture time-varying features.
- This paper proposes an adaptive cross-domain attention mechanism to fuse time-domain and frequency-domain features. This mechanism effectively extracts and integrates key information in time and frequency feature and realizes feature-level interaction and fusion.
- This paper compared and analyzed the proposed model with other deep learning models and mainstream methods based on the same dataset and enhanced the transparency and interpretability of the model by the SHAP method, thus verifying the superior performance and application potential of the model in the field of autism EEG signal diagnosis.

## 2. Materials and Methods

### 2.1. Dataset

This paper utilized the King Abdulaziz University dataset and the University of Sheffield dataset to include diverse age groups and ensure sample variety.

#### 2.1.1. King Abdulaziz University Dataset

The KAU dataset [32] used in this paper is public and can be obtained by researchers through the link (https://malhaddad.kau.edu.sa/Pages-BCI-Datasets.aspx (17 October 2024)) or by contacting the dataset owner, Dr. Mohammed Jafar Alhaddad, for a request via email (malhaddad@kau.edu.sa). The dataset does not contain the participants’ personal identification information to ensure their privacy. The dataset includes 17 subjects: 12 ASD participants (boys and girls aged 6–20 years) and five controls (boys aged 9–13 years). For more information about the dataset, please refer to [28]. In addition, to keep the data set balanced and prevent overfitting, we visually selected five cases of data with good artifact-free from the ASD category to maintain the balance with the control group.

#### 2.1.2. The University of Sheffield Dataset

Another dataset used in this paper is the Sheffield University Public Dataset [33]. It includes data from 28 individuals diagnosed with Autism Spectrum Disorders (ASDs) and 28 neurologically normal controls, with participants ranging in age from 18 to 68 years. The recordings were made during 2.5 min (150 s) of rest with eyes closed. In addition, the authors re-referenced the data as the average of all electrodes, with the data collected at a sampling frequency of 512 Hz. Data collection and sharing were permitted with permission from the Health Research Authority, specifically under IRAS ID 212171 [33].

### 2.2. Preprocessing

The raw EEG signals went through several different preprocessing stages. First, we downsampled the data to 256 Hz. Subsequently, a Butterworth bandpass filter (0.5–100 Hz) was used to remove noise and high-frequency components of nonbrain electrical activity from the EEG signals. We then decomposed the preprocessed the EEG signals using independent component analysis (ICA) to identify and remove components related to non-neural artifacts such as eye movements, electrocardiograms, and electromyography. In addition, a whole-brain average reference was applied to standardize the signals across channels. Furthermore, we applied 50 Hz and 60 Hz notch filters to the KAU dataset and the University of Sheffield dataset, respectively, to remove line noise. Finally, we performed Z-score normalization on the preprocessed datasets.In this paper, the preprocessed dataset was divided into nonoverlapping signal segments, with each segment lasting 2 s as a sample.

### 2.3. Overview of Time–Frequency Synergy Network Architecture

**Figure 1:**
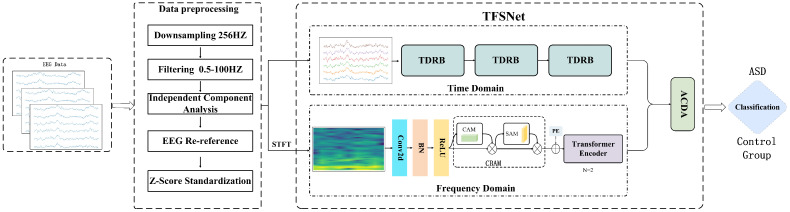
Overall visualization of the time–frequency synergy network (TFSNet) architecture.

To more comprehensively characterize the feature information in EEG signals, this paper proposes a time–frequency synergy network—TFSNet—which integrates time-domain and frequency-domain features. The main process and architecture are shown in Figure 1. As shown, the TFSNet consists of two streams—a time-domain stream and a frequency-domain stream—which extract and fuse time-domain and frequency-domain features, respectively. The time-domain stream extracts the time characteristics of the signal through a dynamic residual network constructed by dynamic convolution, while the frequency-domain stream first uses Short-Time Fourier Transform (STFT) to obtain the frequency domain characteristics. To achieve efficient interaction and fusion between the two features, this paper also proposes an adaptive cross-domain attention mechanism to further improve the overall performance of the model. By combining time-domain and frequency-domain features, the model effectively captures multi-scale features and global patterns in EEG signals, enhancing the accuracy and robustness of Autism Spectrum Disorder (ASD) classification. The design and implementation of the model are described in detail below.

#### 2.3.1. Temporal Feature Extraction with Dynamic Convolution and Residual Connection

**Figure 2:**
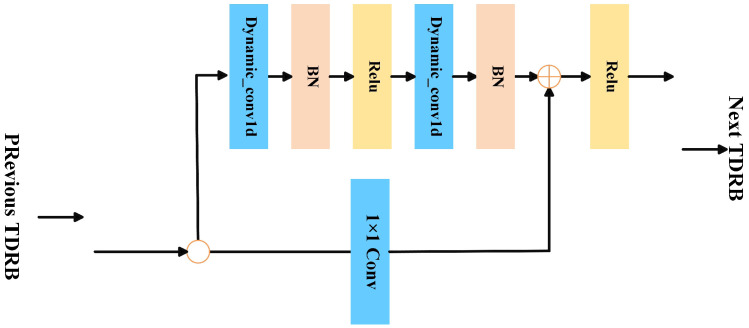
Structure of the Time-Domain Dynamic Residual Block (TDRB).

Dynamic convolution, unlike static convolution with fixed kernels, adaptively generates convolution kernels for each input, improving temporal feature extraction in EEG signals [34]. Therefore, we propose a Time-Domain Dynamic Residual Block (TDRB) that combines dynamic convolution and residual connection mechanisms [35]. The TDRB structure is illustrated in Figure 2. It adaptively adjusts convolution kernel parameters and prevents gradient vanishing, thereby enhancing the model’s sensitivity to different input signals. This improves the model’s ability to extract temporal variations in autism EEG.

Each input feature map $X\in R^{C\times L_{\mathrm{in}\;}}$ of the TDRB first passes through two dynamic convolutional layers, which are each followed by a batch normalization layer (BN) [36] to obtain normalized data. The input features are not only nonlinearly mapped by the dynamic convolution module but also directly connected to the output through identity mapping. The final output $Y\in R^{C\times L_{\mathrm{in}\;}}$ of the dynamic residual block is expressed as

(1) $$Y_{TDRB}=\mathrm{ReLU}\left(B_{2}\left(\left(\mathrm{ReLU}\left(B_{1}\left(\mathrm{Dyconv}1d_{1}\left(X\right)\right)\right)\right)\right)+X_{\mathrm{residual}\;}\right)$$

where

(2) $$X_{\mathrm{residual}\;}=\left\{\begin{array}{c} X,\;C_{\mathrm{in}\;}=C_{\mathrm{out}\;}\\ \mathrm{Conv}1d\left(X;W_{r},b_{r}\right),\;C_{\mathrm{in}\;}\neq C_{\mathrm{out}\;} \end{array}\right.$$

**Figure 3:**
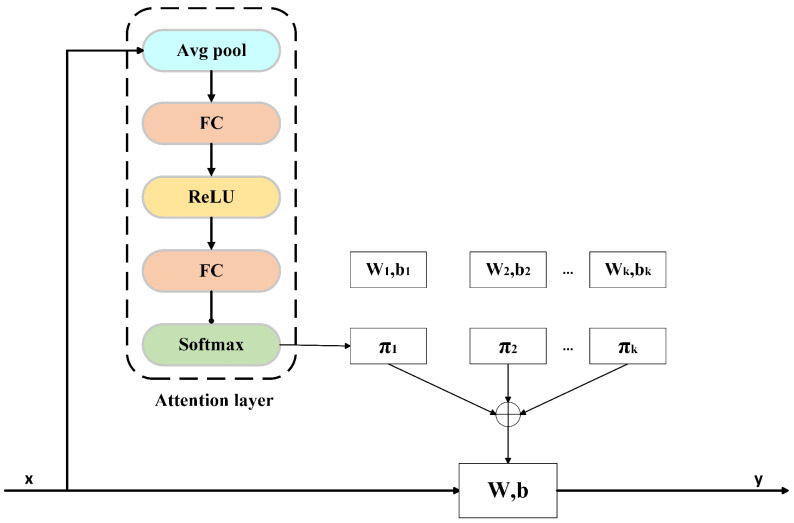
Dynamic convolution calculation process.

$X_{\mathrm{residual}\;}$ is defined based on channel consistency: If the input and output channels match, $X_{\mathrm{residual}\;}=X$; otherwise, $X_{\mathrm{residual}\;}=\mathrm{Conv}1d\left(X;W_{r},b_{r}\right)$, where $W_{r}$ and $b_{r}$ denote the weights and bias of the 1×1 Conv. Here, $Dyconv1d_{i}$ indicates the *i*-th dynamic convolution. Dynamic convolution creates a convolution kernel tailored to the input sample by weighted summation of multiple kernels, enabling a single convolution operation. Compared to traditional convolution, dynamic convolution enhances both feature extraction and representation capabilities. The calculation process is shown in Figure 3, where the input is *x*, and the output is *y*, which are defined as

(3) $$y=W_{\left(x\right)}+b_{\left(x\right)}$$

where

(4) $$W_{\left(x\right)}=\sum_{k=1}^{K}\theta_{k}\left(x\right)W_{k}\left(x\right)$$

(5) $$b_{\left(x\right)}=\sum_{k=1}^{K}\theta_{k}\left(x\right)b_{k}\left(x\right)$$

where $\theta_{k}$ represents the normalized attention weight for the *k*-th kernel (0≤θ≤1, $\sum_{k=1}^{K}\theta_{k}=1$), reflecting its relative contribution to the input *x*. $W_{k}\left(x\right)$ and $b_{k}\left(x\right)$ denote the aggregated kernel weight and bias.

The weight coefficient $\theta_{k}$ in the dynamic convolution is not fixed but is adjusted accordingly as the input EEG signal changes dynamically. It is determined by an attention mechanism. This attention mechanism is similar to the Squeeze-and-Excitation attention mechanism (SE) [37], except that it uses softmax instead of sigmoid as the activation function, which can dynamically adjust the output sparsity. The calculation process can be expressed as

(6) $$z=GAP\left(x_{k}\right)$$

(7) $$s_{1}=\mathrm{ReLU}\left(W_{1}z+b_{1}\right)$$

(8) $$s_{2}=W_{2}s_{1}+b_{2}$$

(9) $$\theta_{k}=\frac{\exp\left(s_{k}/\tau \right)}{\sum_{k=1}^{K}s_{j}/\tau }$$

where $GAP(x_{k})$ denotes the global average pooling operation. $s_{j}$ is the unnormalized attention score of the *j*-th convolution kernel, which is used to calculate the final attention weight, and $s_{k}$ represents the unnormalized attention score of the *k*-th convolution kernel. A constant τ controls the smoothness of these weights, ensuring that the early attention distribution remains nearly uniform.

#### 2.3.2. Attention-Enhanced Frequency-Domain Feature Extraction

Frequency-domain features offer a unique perspective on neural activity and can reveal the specific functions of different frequency bands in EEG signal analysis. Furthermore, time-domain and frequency-domain features complement each other [38]. Leveraging both features can significantly enhance EEG signal decoding accuracy. Therefore, based on the time-domain feature extraction, in this paper, we adopt a frequency-domain feature extraction branch and integrate it with the time-domain feature fusion strategy to improve the overall performance of the model. In addition, frequency-domain features also are extensively applied in the research and diagnosis of neurological disorders, such as attention deficit hyperactivity disorder (ADHD) [39] and epilepsy detection [40].

Prior to feature extraction, we apply the Short-Time Fourier Transform (STFT) to the raw EEG signals to capture features in frequency domains. In this paper, the Short-Time Fourier Transform adopts the Hanning window, with a window length of 256 and an overlap of 50%. Next, a convolutional neural network (CNN) processes the frequency-domain features to extract low-level local features, capturing local patterns and dynamic changes. To further enhance the characteristics of the frequency domain, extract key information, and suppress irrelevant characteristics, a Convolutional Block Attention Module (CBAM) [41] is introduced in the branch of the frequency domain. The CBAM consists of two components: channel attention and spatial attention. Its primary function is to adaptively assign higher weights to important features, enhancing the model’s representational capacity. In this paper, the CBAM adopts the standard configuration, including the sequential combination of two sub-modules: channel attention and spatial attention.

When extracting features in the frequency domain of EEG signals, in addition to focusing on the local features of the signal, it is also necessary to capture long-term dependencies and global patterns between different periods. To capture global patterns and long-range dependencies in frequency-domain features, a Transformer encoder is introduced after the Convolutional Block Attention Module (CBAM) to further model the spectral features, thereby enhancing the model’s classification ability for Autism Spectrum Disorder (ASD). In this paper, we only used the Transformer encoder part, adopted four parallel attention heads, and connected the Transformer encoder part to the Convolutional Block Attention Module (CBAM) to model the global state, thereby capturing the global dependencies of the EEG.

#### 2.3.3. Time-Frequency Feature Fusion of Adaptive Cross-Domain Attention Mechanism

In autism EEG data, the features in the time and frequency domain are highly complementary. However, they are relatively independent in their feature representation. Simply concatenating the two features may introduce redundant or irrelevant information, hindering effective model learning [42,43]. Therefore, this paper proposes an Adaptive Cross-Domain Attention mechanism (ACDA), which facilitates interaction and fusion between time- and frequency-domain features and improves the decoding accuracy of EEG signals.

**Figure 4:**
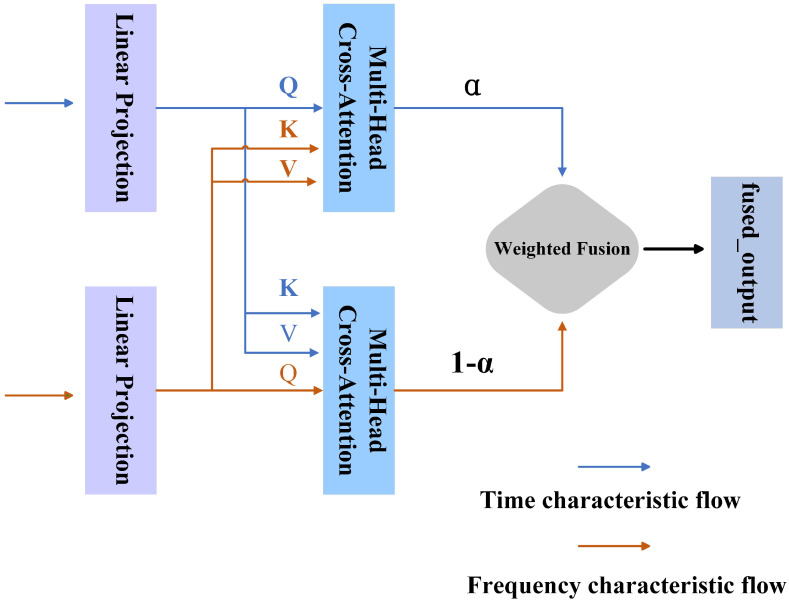
Adaptive Cross-Domain Attention mechanism structure.

Unlike traditional multi-head attention mechanisms that typically focus on multi-view learning within the same feature domain, ACDA emphasizes cross-domain interactions between time-domain and frequency-domain representations. These attention heads operate in different directions (time-to-frequency and frequency-to-time) to capture complementary information and simultaneously suppress redundant signals. Among them, the multi-head attention mechanism can be understood as the process in which the model analyzes EEG data from multiple perspectives simultaneously, similar to how clinicians integrate different types of information (such as behavioral observations and neural signals) to form a comprehensive diagnosis. Each “head” in this mechanism focuses on a specific aspect of the time–frequency relationship, enabling the model to capture complex neural patterns related to ASD classification. Compared to more complex fusion techniques (e.g., bilinear pooling), ACDA avoids excessively increasing model parameters while still capturing higher-order correlations across domains. The specific structure is illustrated in Figure 4, where multiple attention heads adaptively learn to emphasize the most discriminative features for robust ASD classification.

The input features $X_{t}$ and $X_{f}$ are first linearly projected into their respective query, key, and value matrices: $Q_{t}$, $K_{t}$, $V_{t}$ for the time domain and $Q_{f}$, $K_{f}$, $V_{f}$ or the frequency domain. The cross-domain attention mechanism computes attention weights by interacting the projected matrices. Specifically, $Q_{t}$ from the time domain is matched with $K_{f}$ from the frequency domain to calculate $A_{t\to f}$, representing the attention flow from the time to the frequency domain. Conversely, $Q_{f}$ and $K_{t}$ compute $A_{f\to t}$, reflecting attention flow in the opposite direction. These computations effectively model the interplay between the time and frequency domains, as formalized below:

(10) $$A_{i\to j}=\mathrm{softmax}\left(\frac{Q_{i}K_{j}^{⊤}}{\sqrt{d_{k}}}\right),\;i,j\in \left\{t,f\right\},i\neq j$$

where $A_{t\to f}$ and $A_{f\to t}$ denote the attention weights between the time and frequency domains in both directions. To enhance adaptability, learnable weight parameters $W_{t}$ and $W_{f}$ are introduced to adjust these weights, producing the weighted results dynamically.

(11) $$A_{i\to j}^{\prime }=A_{i\to j}\times W_{i},\;i,j\in \left\{t,f\right\},i\neq j$$

After obtaining the weighted attention weights, we further calculate the output of the cross-domain features:

(12) $$O_{i}=A_{i\to j}^{\prime }\times V_{j},\;i,j\in \left\{t,f\right\},i\neq j$$

where $O_{t}$ represents the time domain feature output obtained by weighting the attention from the time domain to the frequency domain, and $O_{f}$ represents the frequency domain feature output obtained by weighting the attention from the frequency domain to the time domain.

To reduce the feature redundancy after fusion, we use an adaptive fusion factor α to perform a weighted fusion of the two features $O_{t}$ and $O_{f}$ to obtain the final fusion feature *O*. The formula is as follows:

(13) $$\alpha =\sigma \left(W_{\alpha }O_{t}+b_{\alpha }\right)$$

(14) $$O=\alpha O_{t}+\left(1-\alpha \right)O_{f}$$

where σ represents the sigmoid activation function, and $W_{\alpha }$ and $b_{\alpha }$ are learned parameters. Through this fusion strategy, the model can automatically adjust the fusion ratio of time domain and frequency domain features according to the specific distribution of the data, thereby optimizing the joint expression of features.

### 2.4. Performance Evaluation Parameters

We uses different evaluation metrics to evaluate our proposed model, including accuracy, precision, recall, specificity, and F1-score.

(15) $$\;\mathrm{Accuracy}\;=\frac{TP+TN}{TP+TN+FP+FN}$$

(16) $$\;\mathrm{Precision}\;=\frac{TP}{TP+FP}$$

(17) $$\;\mathrm{Recall}\;=\frac{TP}{TP+FN}$$

(18) $$\begin{array}{c} \;\mathrm{Specificity}\;=\frac{TN}{TN+FP} \end{array}$$

(19) $$\;F1-\mathrm{score}\;=\frac{2*TP}{2*TP+FP+FN}$$

*TP* and *TN* are true positive and true negative, respectively, and *FP* and *FN* are false positive and false negative, respectively.

## 3. Results

### 3.1. Model Parameters

To evaluate the performance of the model, we randomly divided the EEG data into five equal parts, used 5-fold cross-validation, and obtained the final average evaluation index. During the training process, the batch size and the number of training rounds (epochs) were set to 32 and 100, respectively, the initial learning rate was 0.01, and the Adam optimizer was used. There are two key parameters K and τ in the dynamic convolution. To effectively determine the optimal values of these two parameters, we adopted a grid search strategy. For each set of parameter combinations, the same training configuration was used for experiments, and the best parameter combination was selected based on the minimum loss value on the test set. The experimental results show that when K and τ are 10 and 25, respectively, the model performance is optimal. Therefore, this set of parameter settings was used in the final model training to ensure the best feature extraction effect and classification performance.

### 3.2. Experimental Results and Analysis

This section analyzes and summarizes the results of the classification experiments conducted on the University of Sheffield dataset and the KAU dataset. The experimental results show that TFSNet has significant effectiveness in feature extraction, as shown in Table 1 and Table 2. On the University of Sheffield dataset, the accuracy of Random Forest (RF), K Nearest Neighbors (KNN), and Support Vector Machine (SVM) were all below 85% when used alone, reflecting the limitations of traditional methods in feature extraction. However, when combined with the features extracted by TFSNet, the overall accuracy of the classifiers was over 97%, with the combination of RF and TFSNet achieving an accuracy of 99.27%. On the KAU dataset, the accuracies of RF, KNN, and SVM alone were all below 90%, whereas when combined with TFSNet, the accuracies of all combinations were above 97%. This indicates that the highly discriminative features extracted by TFSNet through dynamic convolution and time–frequency feature fusion effectively compensate for the limitations of traditional classifiers and significantly improve classification accuracy.

In addition, TFSNet also performed well compared to mainstream deep learning models on both datasets, as shown in Table 3 and Table 4. In the results of the deep learning comparison experiments, on the University of Sheffield dataset, the accuracy results of EEGNet, DeepConvNet, and ShallowConvNet were below 95%, while TFSNet reached 98.68%. On the KAU dataset, the accuracy results of EEGNet, DeepConvNet, and ShallowConvNet were are all below 90%, and the other methods such as Tawhid et al. [26] and Ullah et al. [31] yielded 95.25% and 93.56%, respectively, while the accuracy of TFSNet was 97.14%, which outperforms all the comparative models and shows its superiority in processing complex data patterns. This indicates that TFSNet has strong feature extraction and generalization ability in complex data pattern processing and can effectively capture the main features of EEG signals in different data sets, thus improving the classification performance. To ensure a comprehensive evaluation, we report the variance from the 5-fold cross-validation in this study. For the University of Sheffield dataset, the variance in accuracy is 0.55%, and for the KAU dataset, it is 0.7%. These low variances suggest that the model’s performance is stable across different folds, reinforcing the reliability of the reported results.

It can be seen from Table 2 and Table 4 that the performance of the model on the KAU dataset was not satisfactory. This may stem from inherent differences in EEG signals between adolescents and adults, as the KAU dataset consists of adolescent subjects, while the Sheffield dataset consists mainly of adults. Adolescent brain activity patterns may exhibit greater variability or different frequency characteristics that are not fully captured by the model. In addition, differences in data collection methods or devices between the two datasets may have introduced subtle variations that affected performance.

### 3.3. Dynamic Convolutional Attention Weight Visualization

**Figure 5:**
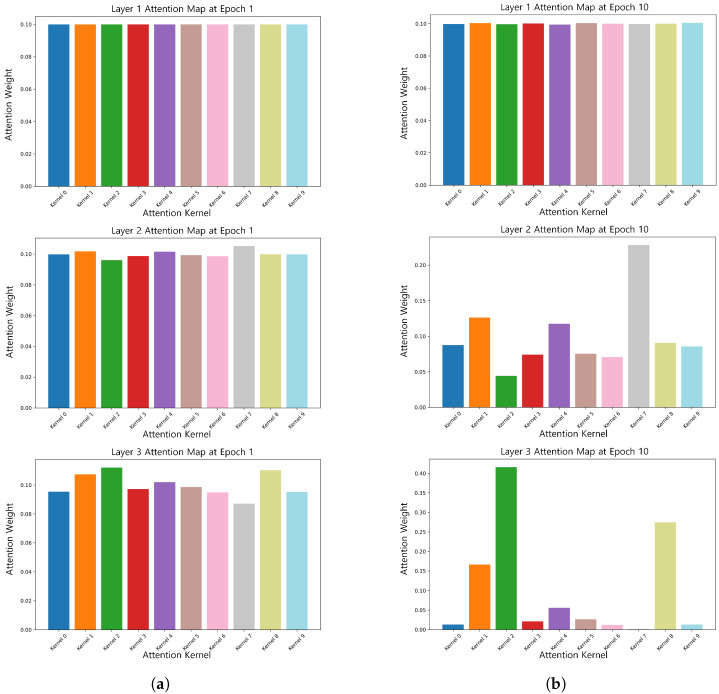
Attention weights of the same subject at epoch 1 (τ=25) and epoch 10 (τ=1). (**a**) Attention weights of the same subject at epoch 1 (τ=25). (**b**) Attention weights of the same subject at epoch 10 (τ=1).

To verify the effectiveness of dynamic convolution in dealing with the time-varying nature of EEG signals, we visualized its attentional weights, as shown in Figure 5. Figure 5 shows that in the early stage of training (1st epoch), the attention weights of each convolutional kernel were more uniformly distributed due to the large temperature coefficient τ, which allowed the model to fully explore the features in the early stage. As training progressed, τ gradually decreased, and the attention weights began to focus on key features. For example, by the 10th epoch, the attention of some of the convolutional kernels in the second layer decreased, while the attention of the third layer was focused on kernel 1, kernel 2, and kernel 8.

**Figure 6:**
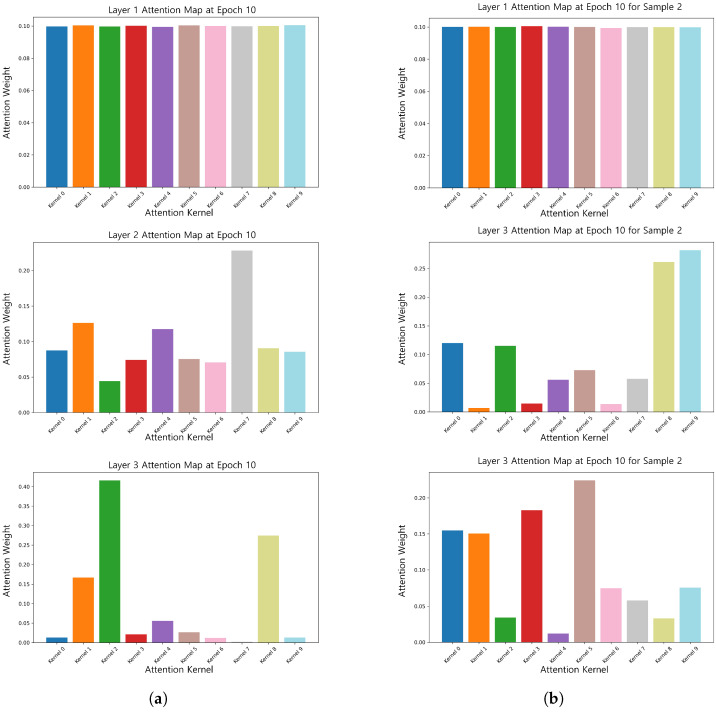
Attention weights of the 10th epoch for different samples (epoch = 10). (**a**) Attention weight map of sample 1 at epoch = 10. (**b**) Attention weight map of sample 2 at epoch = 10.

Furthermore, the attentional weights of different samples dynamically convolved at the 10th epoch (τ=1) were visualized, as illustrated in Figure 6. The results indicate that for the temperature coefficient, the attentional weights of different samples exhibited differences. Specifically, one sample allocated more attention to the features of kernel 1, kernel 2, and kernel 8, while the other sample focused on other key features. This phenomenon suggests that dynamic convolution can dynamically adjust the attention weights according to the characteristics of the input samples, gradually shifting from global features to sample-specific key features, thus improving the model’s ability to extract complex signal features.

### 3.4. Interpretability Analysis Based on SHAP

**Figure 7:**
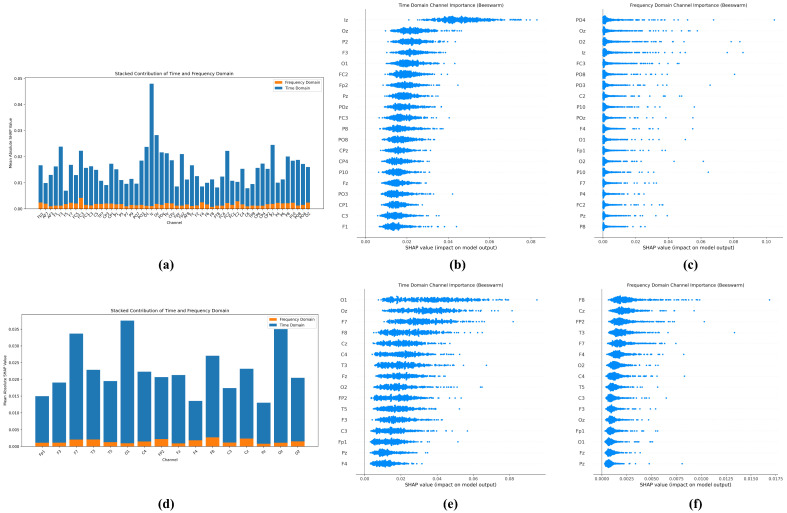
Explainable analysis results based on SHAP: (**a**–**c**) are the analysis results of the University of Sheffield dataset; (**d**–**f**) are the analysis results of the KAU dataset.

Figure 7 shows the interpretability analysis of the TFSNet model performed by the SHapley Additive exPlanations (SHAP) method, aiming to reveal the contribution patterns of electrodes in different brain regions in the classification of Autism Spectrum Disorders (ASDs). This analysis not only validates the model’s classification decisions but also provides important insights into the neurophysiological mechanisms of ASD. For the Sheffield dataset (adult), SHAP analysis showed that electrodes in the occipital region (such as Oz, O1, and O2) exhibited the highest SHAP contribution values in both the time and frequency domains, with the average contribution significantly exceeding the other regions. This result is highly consistent with the findings of clinical studies on abnormalities in visual processing and visual information integration in ASD [46,47]. In addition, the high contribution of electrodes in the parieto-occipital region, such as PO3, PO4, and POz, further highlights the critical role of visual–spatial processing networks in ASD classification. The beeswarm plot in the picture clearly demonstrates the importance of these electrodes, while the contribution of the other electrodes is relatively low, indicating the dominant role of visually related brain areas in adult ASD classification.

Unlike the Sheffield dataset, SHAP analysis of the KAU dataset (adolescents) showed a higher contribution from prefrontal electrodes (e.g., F7, F8, Fp1, and Fp2), which may reflect more significant executive function deficits in adolescents with ASD [48]. Furthermore, the involvement of electrodes in the central region, such as Cz, indicates the importance of sensor motion processing in this population. The difference in the pattern of electrode importance between the two datasets clearly reveals that ASD may present different neurophysiological features at different developmental stages. Overall, the analysis in Figure 7 not only validates the rationality and biological interpretability of the classification decisions made by the TFSNet model but also provides valuable insights for clinical practice. For example, the identified key electrode regions can be used to optimize EEG acquisition protocols and guide personalized diagnostic methods and potential intervention strategies, thereby improving the accuracy and efficiency of ASD diagnosis.

### 3.5. Ablation Experiment

To evaluate the contribution of each component in TFSNet to model performance, we first conducted single-branch ablation experiments on the University of Sheffield and KAU datasets, and the results are shown in Table 5 and Table 6. The experimental results show that the single branch results were lower than TFSNet in both datasets. This paper also conducted ablation experiments on various components of TFSNet, and the results are shown in Table 7 and Table 8. The experimental results show that when only the dynamic convolution in the time domain was removed, the accuracy of the model on the two datasets was reduced by 1.17% and 0.9%, respectively, showing a small performance decline, while the adaptive span in the frequency domain was removed. When removing the Adaptive Cross-Domain Attention mechanism (ACDA), the accuracy dropped most significantly, dropping by 2.33% and 3.1%, respectively, being especially notable on the KAU dataset. In addition, the deletion of the CBAM module decreased the second, with a decrease of 1.32% and 1.36%, respectively. Notably, in the “only time” experiment, the model specificity index using only temporal branches was higher than TFSNet on both datasets, but the recall was lower, which may lead to the risk of missed diagnosis and hence delayed intervention timing.

The above experimental results verify our hypothesis that dynamic convolution can adaptively adjust the convolution kernel and effectively improve the ability to capture the temporal variation characteristics of different EEG signal features. It is worth noting that the Adaptive Cross-Domain Attention mechanism (ACDA) plays a key role in the interaction and fusion of time–frequency features, significantly enhancing the performance of the model. In addition, the CBAM module can also better enhance key features in the frequency domain and suppress uncritical features, thereby further improving the robustness and generalization ability of the model.

## 4. Discussion

This paper proposes a time–frequency synergy network (TFSNet) for Autism Spectrum Disorder (ASD) classification using EEG signals, and it was validated on the University of Sheffield and KAU datasets.

TFSNet introduces an innovative architecture that addresses these challenges by integrating time- and frequency-domain features. Using dynamic convolution, TFSNet adaptively adjusts convolution kernels to capture the time-varying characteristics of EEG signals, overcoming the limitations of static convolution in models like EEGNet. In the frequency domain, it employs Short-Time Fourier Transform (STFT) and a Convolutional Attention Module (CBAM) for robust feature extraction. The Adaptive Cross-Domain Attention mechanism (ACDA) enables bidirectional interaction and fusion of time- and frequency-domain features, enhancing classification performance and generalization. This time–frequency synergy makes TFSNet more robust and effective for ASD classification across varied datasets.

TFSNet, in combination with SHAP analysis, revealed key EEG patterns associated with ASD that were highly correlated with known ASD neurophysiological features such as abnormal activity of theta and alpha waves [46,47]. This finding provides clinicians with interpretable diagnostic evidence to help distinguish ASD subtypes and guide treatment decisions. For example, the specific frequency band activity differences identified by TFSNet could be used to design targeted neurofeedback training to improve social or cognitive function in patients. In addition, SHAP analysis identifies the most important electrode locations for diagnosis, which provides the possibility to optimize EEG acquisition, significantly reducing the time and cost of the examination and making it more viable for application in resource-limited clinical Settings. In terms of personalized diagnosis, TFSNet can generate diagnostic markers based on patient-specific EEG patterns, which can provide support for the formulation of individualized treatment plans. For example, in clinical practice, physicians can use the output of TFSNet in combination with other clinical assessments to develop targeted intervention strategies. However, the limitation of the current model is that its validation data are mainly from specific populations, and its generalization ability needs to be further tested in a wider patient population in the future. In addition, the integration of TFSNet into existing clinical workflows may face technical compatibility and ethical challenges, which will be further explored in subsequent studies.

Despite the excellent performance of TFSNet, its limitations cannot be ignored. Firstly, the computational complexity of the model is a key issue. Its multi-component architecture, including dynamic convolutions, CBAM modules, Transformer encoders, and ACDA mechanisms, significantly improves performance but also increases computational cost. In resource-constrained clinical settings, such as using portable EEG devices or within real-time diagnostic systems, this complexity may limit the deployment of models. Future research should explore model compression techniques such as pruning or quantization to reduce computational requirements while maintaining high accuracy. In addition, the real-time performance of the model needs to be further optimized to meet the requirements of rapid diagnosis in clinical environments.

Interpretability is another area that needs to be improved. Although SHAP analysis reveals the importance of features, its interpretation is still biased toward the technical level, which makes it difficult to fully meet the intuitive interpretation needs of clinicians for direct association with functional brain regions. For example, more clarity is still needed on how models relate specific EEG patterns to behavioral phenotypes or treatment effects in ASD. Future work can incorporate neuroscience knowledge to develop visualization tools closer to clinical context to enhance the transparency and credibility of the model in medical decision making.

## 5. Conclusions

In this paper, we propose the time–frequency synergy network (TFSNet), which uses electroencephalogram (EEG) signals for robust Autism Spectrum Disorder (ASD) classification, and has been validated on multiple datasets. TFSNet’s innovative architecture provides promising insights for personalized ASD diagnosis and targeted intervention strategies by collaboratively fusing time- and frequency-domain features and enhancing interpretability through SHAP analysis. However, several future works are still needed to translate TFSNet from research to practical clinical applications. Its current computational complexity needs to be optimized by model compression techniques (e.g., pruning, quantization, etc.) to ensure real-time deployment and hardware feasibility, especially for resource-constrained portable EEG systems. Crucially, comprehensive clinical testing across a broader and more diverse patient population via multicenter trials is needed to validate its generalization ability and clinical utility while addressing technical compatibility and ethical challenges for seamless integration into existing healthcare workflows. Overcoming these practical deployment challenges will be key for TFSNet to become a valuable diagnostic tool in clinical settings.

## Figures and Tables

**Table 1:** Performance comparison of traditional classifiers and TFSNet combination on the University of Sheffield dataset.

	Accuracy (%)	Precision (%)	Recall (%)	Specificity (%)	F1-Score (%)
RF	62.79	65.92	58.64	67.28	62.07
KNN	82.35	87.70	76.77	88.38	81.87
SVM	65.35	80.10	44.87	61.76	64.21
RF + TFSNet	99.27	99.20	99.47	99.03	99.33
KNN + TFSNet	98.83	98.41	99.47	98.06	98.94
SVM + TFSNet	98.39	98.40	98.66	98.06	98.94

**Table 2:** Performance comparison of traditional classifiers and TFSNet combination on the KAU dataset.

	Accuracy (%)	Precision (%)	Recall (%)	Specificity (%)	F1-Score (%)
RF	68.24	87.79	44.84	93.32	59.37
KNN	85.40	86.75	62.83	89.72	72.88
SVM	83.25	83.73	83.93	82.52	83.83
RF + TFSNet	97.64	98.17	97.49	97.82	97.83
KNN + TFSNet	97.64	98.39	97.27	98.09	97.82
SVM + TFSNet	97.52	98.16	97.27	98.09	97.82

**Table 3:** Performance comparison of mainstream deep learning models on the University of Sheffield dataset.

	Accuracy (%)	Precision (%)	Recall (%)	Specificity (%)	F1-Score (%)
EEGNet [44]	78.75	78.13	79.67	77.83	78.78
DeepConvNet [45]	88.38	87.52	89.66	87.10	88.54
ShallowConvNet [45]	94.88	95.21	94.57	95.19	94.88
CNN-Lstm	84.14	87.72	80.44	87.86	83.30
Ullah et al. [31]	95.28	92.86	98.11	98.11	95.40
TFSNet	98.68	98.41	99.20	98.06	98.80

**Table 4:** Performance comparison of mainstream deep learning models on the KAU dataset.

	Accuracy (%)	Precision (%)	Recall (%)	Specificity (%)	F1-Score (%)
EEGNet [44]	70.14	70.47	74.59	65.39	72.33
DeepConvNet [45]	88.60	91.49	86.02	91.36	88.64
ShallowConvNet [45]	87.48	89.27	86.17	88.89	87.68
CNN-Lstm	82.49	83.37	82.66	82.30	82.99
Ullah et al. [31]	93.56	94.88	92.31	92.31	93.58
TFSNet	97.14	98.71	94.45	99.29	96.81

**Table 5:** Single-branch ablation experiment on the University of Sheffield dataset.

	Accuracy (%)	Precision (%)	Recall (%)	Specificity (%)	F1-Score (%)
Only time	95.03	98.13	91.84	98.24	94.88
Only freq	92.69	92.22	93.29	92.08	92.75
TFSNet	98.68	98.41	99.20	98.06	98.80

**Table 6:** Single-branch ablation experiment on the KAU dataset.

	Accuracy (%)	Precision (%)	Recall (%)	Specificity (%)	F1-Score (%)
Only time	88.59	99.69	78.18	99.74	87.63
Only freq	91.32	99.15	83.93	99.23	90.91
TFSNet	97.14	98.71	94.45	99.29	96.81

**Table 7:** Ablation results using the University of Sheffield dataset.

	Accuracy (%)	Precision (%)	Recall (%)	Specificity (%)	F1-Score (%)
Without Dynamic_conv	97.51	98.64	96.79	98.39	97.71
Without CBAM	97.36	94.86	100	94.87	97.36
Without ACDA	96.35	97.81	95.45	97.42	96.22
TFSNet	98.68	98.41	99.20	98.06	98.80

**Table 8:** Ablation experiment results using the KAU dataset.

	Accuracy (%)	Precision (%)	Recall (%)	Specificity (%)	F1-Score (%)
Without Dynamic_conv	96.15	98.32	94.17	98.22	96.12
Without CBAM	95.78	99.51	92.71	99.46	95.99
Without ACDA	94.04	93.16	96.13	91.55	94.62
TFSNet	97.14	98.71	94.45	99.29	96.81

## Data Availability

The dataset used for this study is publicly available and accessible online from https://malhaddad.kau.edu.sa/Pages-BCI-Datasets.aspx (17 October 2024) and DOI: https://doi.org/10.1016/j.cortex.2021.09.022.
